# Filling the gap between collection, transport and storage of the human gut microbiota

**DOI:** 10.1038/s41598-019-44888-8

**Published:** 2019-06-06

**Authors:** Noelia Martínez, Claudio Hidalgo-Cantabrana, Susana Delgado, Abelardo Margolles, Borja Sánchez

**Affiliations:** 1 Microviable Therapeutics SL. Edificio Severo Ochoa, Campus de El Cristo. C/Fernando Bonguera s/n, 33006 Oviedo, Spain; 2 Department of Food, Processing and Nutrition Sciences, North Carolina State University, Raleigh, NC 27695 USA; 3 Departamento de Microbiología y Bioquímica, Instituto de Productos Lácteos de Asturias (IPLA), Consejo Superior de Investigaciones Científicas, Asturias, CSIC Spain

**Keywords:** Microbial ecology, Microbiome

## Abstract

Stool collection devices minimizing the exposure of gut bacteria to oxygen are critical for the standardization of further microbiota-based studies, analysis and developments. The aim of this work was to evidence that keeping anaerobiosis has a deep impact on the viability and diversity of the fecal microbiota that is recovered in the laboratory. Recovering certain microbial populations, such as obligate anaerobic bacteria, is particularly critical if the purpose of the study is to envisage personalized therapeutic purposes, such as autologous Fecal Microbiota Transplant. In this study the same fecal specimens were sampled in conventional stool containers and GutAlive, a disposable device that minimizes exposure of the gut microbiota to oxygen. Samples from five healthy donors were analysed and 150 differential colonies were recovered and identified by 16S rRNA gene sequencing. Globally, GutAlive maintained extremely oxygen sensitive (EOS) populations that were lost in conventional stool containers, and thus viability of species such as as *Akkermansia muciniphila*, *Faecalibacterium prausnitzii* and a novel member of the *Clostridiales* order was kept. These obligate anaerobes were not recovered using the conventional stool collection device. In conclusion, the use of GutAlive for stool collection and transport optimized the viability and recovery of EOS bacteria in the lab by diminishing oxygen toxicity.

## Introduction

There is currently a lack of consensus in the methodologies and procedures for the collection and preservation of stool samples, notably if further downstream metagenomic applications are envisaged. Sample manipulation and other related procedures during the first steps of microbiota analysis could be among the most impactful variables, and they have been mishandled systematically during the last years. In 2017, Gohl and colleagues reported the different sources of error and bias during metagenome analysis^1^. Notably, they identified sample collection and storage as two steps that have not been conveniently addressed until now. Different protocols had tremendous impact on the final estimation of microbial populations, as well as on the capability to perform or not, complementary studies such as transcriptomic and metabolomics^2^. Only the step of total DNA extraction applying different protocols yielded different results when applied to a defined bacteria community, highlighting the bias that bacteria lysis (enzymatic, mechanical or both) incorporated into the final results^3^. With this in mind, it seems quite difficult to envisage how the results of microbiome studies using just different sampling methods could be compared.

Variables such as oxygen toxicity during fecal sample collection and transport can be even more impactful if bacterial viability, notably affecting the populations of obligate anaerobes, is considered. Maintaining viability of these extreme oxygen sensitive (EOS) bacteria is a key factor for the designing of microbiota-based therapies, such as autologous or heterologous fecal microbiota transplantation (aFMT or FMT, respectively)^4,5^. To our knowledge, there is no disposable device designed for the maintenance of microbial viability during stool sampling, as DNA stability has been the main focus of attention in the main microbiome projects. The impact of oxygen toxicity is rarely taken into consideration, even if it is well known that many of the EOS bacteria present in the stool samples die after a few minutes of exposure to atmospheric oxygen concentrations, making necessary its manipulation in anaerobic conditions^6^.

Using different stool collection devices and strategies has a deep impact on the viability and diversity of the fecal microbiota that is recovered in the laboratory after sampling and transport. Oxygen toxicity is particularly critical if the purpose of the sample is to envisage personalized therapeutic purposes, such as aFMT or the design of tailored biotherapeutics^4,7^. Generation of an anaerobic environment in the stool container as soon as the lid is closed, is expected to remove the oxygen from the inner atmosphere minimizing the oxygen exposition and toxicity over anaerobic bacteria. In the case of conventional stool sampling devices, which do not contemplate avoiding oxygen toxicity, one should expect that dominant and meaningful EOS bacteria such as *Faecalibacterium* sp. will be dead after few minutes of exposure to atmospheric oxygen concentrations^8^.

In this paper we present GutAlive, a stool collection kit for fecal sampling and transport that ensures the viability of the fecal microbiota, including obligate anaerobes and EOS bacteria. This product follows the same principle of an anaerobic system but in the form of a portable, single use device, which generates an anaerobic atmosphere just right after stool collection. This approach minimizes oxygen exposition and ensures the anaerobic conditions during sample transport and delivery. The use of GutAlive in protocol normalization and downstream applications are discussed in the manuscript.

## Results and Discussion

Sample collection device and conditions, the donor manipulation, the temperature of preservation of the sample, the formulation stabilization buffer for nucleic acids, and the time between sample collection and laboratory analyses are the main variables that can influence the miscalculation of the microbial populations present in the stool sample. Most importantly, keeping anaerobic conditions from the sampling moment is expected to have a positive impact on the recovery of obligate anerobe or EOS bacteria.

**Figure 1:**
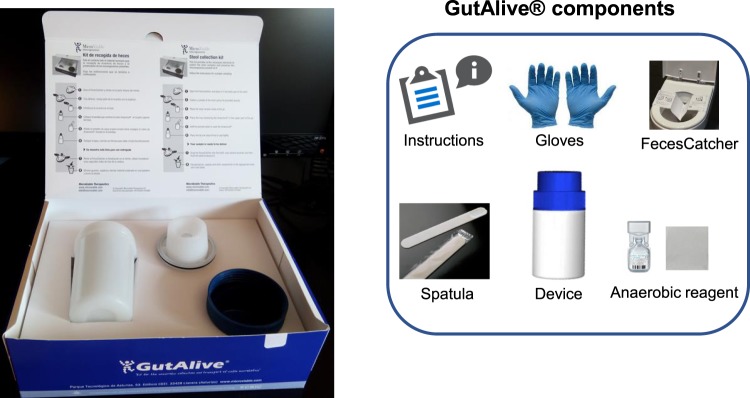
GutAlive, stool collection kit for fecal sampling and transport and its components.

GutAlive is delivered as a kit, containing all the necessary components to facilitate the process of stool collection (Fig. 1). Noteworthy, the use of GutAlive allows a period of 24–48 hours in which the feces can be sampled and delivered to the laboratory at room temperature without significant reduction of bacterial viability. This is a considerable improvement for normalization purposes in large studies involving sampling in different geographic locations, as the viability of the sample is warranted for the standard service offered by many courier companies. The stability at room temperature (RT) is expected to improve and enhance the current protocols used for FMT/aFMT that up to day reflects a lack of consensus and sometimes includes frozen the sample in home freezers^4,5^. For a better understanding on the improvements offered by GutAlive, all the results presented in this work are compared with the conventional device widely used for stool sampling.

### Grown and survival of obligate anaerobes and EOS bacteria

**Figure 2:**
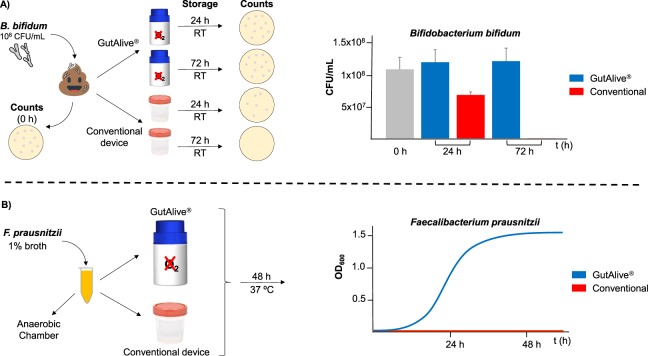
(**A**) A liquid culture of *Bifidobacterium bifidum* was added to stool sample and incubated in a conventional stool sampling device or in GutAlive to assess survival capability by counts (CFUs/mL) after 24 h and 72 h. (**B**) *Faecalibacterium prausnitizii* was inoculated in culture broth and incubated in the conventional stool sampling device or GutAlive to monitor growth (OD_600_) over 48 h. Experiments were performed in independent triplicates.

Initially, we analyzed the effect of the two collection devices (GutAlive vs conventional stool sampling device) on the viability of two well-known anaerobic bacteria. *Bifidobacterium bifidum* LMG11041 is a representative obligate anaerobe naturally present in the healthy human microbiota. This bacterium was added to a fecal sample (previously sterilized twice by autoclaving 15 min at 121 °C) at a concentration 10^8^ cfu/mL. The fecal sample was split in the two collection devices and incubated at RT for 24 h and 72 h. Then, fecal dilutions were made and counts were performed on MRSc plates (Fig. 2A). Overall, GutAlive was able to maintain the viability of *B. bifidum* LMG11041 over 90% during the 72 h compared to the initial time point (control, t = 0 h), whereas in the conventional device the viability was 66% during the first 24 h with no bacteria recovery after 72 h.

In the case of *Faecalibacterium prausnitzii*, a representative species of EOS bacteria, we monitored the growth kinetics of the strain M21/2^9^ in liquid medium as the increase in the turbidity (OD_600_). Screw-capped 1.5 mL tubes were inoculated at 1% (v/v) using an active culture and incubated in the two fecal collection devices. GutAlive was able to support growth (OD_600_~1.5) of *F. prausnitzii* M21/2, whilst no growth was observed in the conventional stool collection device (Fig. 2B). The growth curve of M21/2 strain in GutAlive was similar to that obtained in an anaerobic workstation (data not shown). Overall, these two assays probed that GutAlive was able to generate a convenient anaerobic atmosphere that support obligate anaerobes survival and growth.

### Fecal microbiota viability and diversity

As GutAlive showed to be efficient for handling obligate anaerobe/EOS bacteria, we investigated whether this device is able to maintain the microbiota viability during the sampling and delivery process, the first two steps in most microbiome studies.

**Figure 3:**
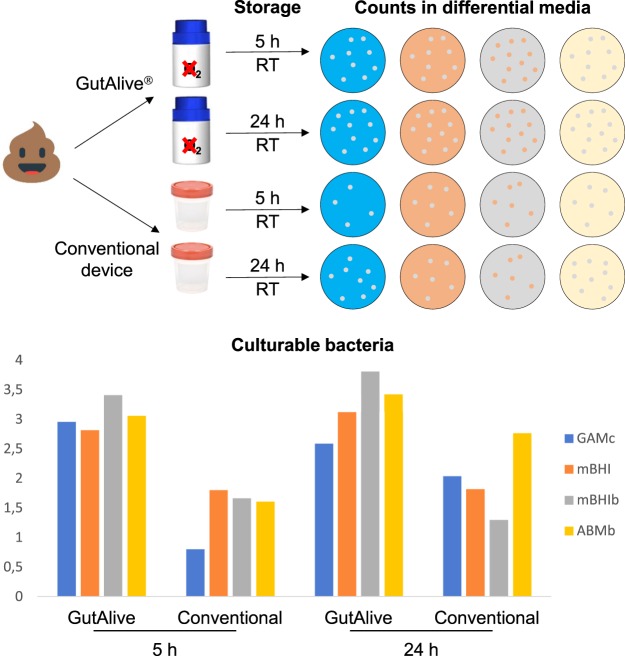
Effect of the use of GutAlive for the collection and sampling of stool specimens in culturable bacteria recovery using four different media after 5 and 24 h from the sampling moment. Results are compared to the use of the conventional stool sampling devices, and diversity is expressed as the Margalef Species Richness.

In order to analyze differences in the viability of fecal microbial populations during the whole transport/delivery procedure, the same fecal specimens were sampled in both conventional stool sampling devices and GutAlive. Samples were kept at RT or 4 °C during 5 h and 24 h, being 5 h considered as time zero of analysis. After these periods both devices were opened into an anaerobic workstation and serial dilutions were made on Maximum Recovery Diluent and spread onto the surface of 4 different pre-reduced media GAMc, mBHI, mBHIb, and ABMb: (Fig. 3). GutAlive device was able to prevent the loose of culturable bacterial diversity observed in traditional containers, even after 24 h, keeping the diversity index around 2.5–3.8 depending on the growth media considered. In contrast, the conventional stool collection device showed markedly lower values of the bacterial diversity index (1.3–2.8 at 24 h; Fig. 3).

The reduction on bacterial diversity using the conventional stool collection device can be correlated with the presence of oxygen that kills obligate anaerobes, whilst favoring the survival of facultative anaerobic/aerotolerant microorganisms. The toxicity of oxygen to dominant microbial populations has been extensively studied in the last years. In this regard, Albenberg and co-workers demonstrated a gradient distribution of microbiota members correlated to oxygen perfusion from gut tissues into the lumen, and showed a higher prevalence of oxygen-tolerant Proteobacteria and Actinobacteria species associated with rectal mucosa^10^. Similarly, aerotolerant microorganisms increased during intestinal inflammation due to a higher presence of oxidative metabolites and oxygen in highly hydrated feces in diarrhea^11^. Noteworthy, Inflammatory bowel disease (IBD) is characterized by a signature dysbiosis of the microbiota where obligate anaerobes are extremely reduced favoring the increase of facultative anaerobes, e.g. decrease in *F. prausnitzii* and increase in *Escherichia coli*, due to the higher presence of oxygen^12^.

**Figure 4:**
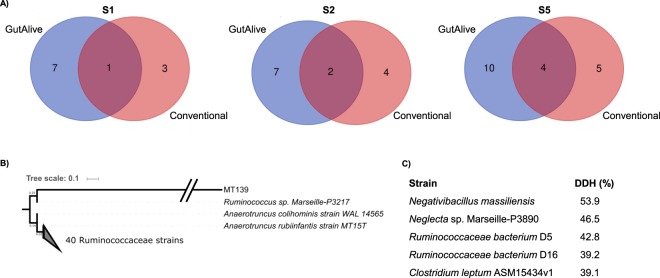
(**A**) Venn’s diagrams showing the different bacteria species isolated in BHI supplemented with defibrinated horse blood after 24 h of sampling. Results from 3 donors (S1, S2 and S5) with different microbiotas are shown. Intersection denotes common isolates identified with both devices. (**B**) Bootstrap consensus NJ tree inferred from 5000 replicates using 16S rRNA gene data from the 42 closest hits to MT139 isolate Retrieved from the GenBank database. Only the three closest hits are shown, and the rest of branches corresponding to the other thirty nine sequences are collapsed. Bootstrap support is shown next to the branches. (**C**) In silico DNA-DNA hybridization values of the closest bacteria to MT139 isolate.

As a proof of concept, we underwent 16S rDNA sequencing of all the colonies isolated from the fecal samples of three healthy donors, using both stool collection devices and sequencing differential and common colonies. In this case, we identified a total of 150 colonies growing in ABMb medium from the three fecal specimens. Differences in colonies were associated to the two different collection devices and also to the individuals (Suppl. File 1, colonies yielding the same sequence are not shown). The microbial diversity, based on the 16S rDNA sequencing data was notably higher using GutAlive device, compared to the diversity obtained with the conventional stool collection device (Fig. 4A). Noteworthy, GutAlive was able to keep the viability of fecal EOS species such as *Akkermansia muciniphila*, *F. prausnitzii* and a possible novel member of the *Clostridiales* Order, among other obligate anaerobes, which were lost using the conventional stool collection device (Suppl. File 1). Notably, the *F. prausnitzii* isolate recovered with GutAlive (MT126) clustered with a subset of other genomes of the same species in a homogeneous group that may represent a separate species given the *in silico* DNA-DNA hybridization values (>70%) obtained from the genomic sequences (Suppl. Fig. 1, Suppl. File 2). GutAlive allowed culturing a novel isolate (MT139) that according to the dilution in which it appeared denoted an abundant isolate, which in addition is a potential butyric acid producer (Fig. 4B, Suppl. File 3). This also demonstrated the importance of a personalized approach in microbiota-based therapies, as this novel *Clostridiales* isolate, which is taxonomically close to the *Ruminococcaceae* family, was only identified in fecal sample of one donor, S5.

Cooling, freezing or even delivering stool specimens at RT have been different approaches applied to preserve DNA for further isolation and subsequential analysis^13^. To test the effect of the temperature on bacterial viability, we firstly tested transport at 4 °C or RT. Temperature did not affect the microbial diversity calculated as the differences in the colony morphologies obtained after sampling, and therefore we kept the most convenient and economic option, RT. Generally, we can affirm that fecal viability and diversity remains intact after 24 h of sampling and store RT when GutAlive is used, as EOS and even novel anaerobic taxa are cultured after this period. We are conscious that the results presented here are limited to the bacteria recovered in a non-selective and rich medium. Higher numbers of gut bacteria would be recovered combining GutAlive with culturomics approach^14^.

Fecal microbiota viability is a key factor to develop microbiota-based products, such as FMT, aFMT and personalized fecal microbiota-based consortia. The generation of microbiota-based products based on viable microbiota will allow developing a whole next generation of personalized therapies to reshape the human microbiome towards healthy configurations. Moreover, as the research on next generation probiotics is currently progressing, defined and personalized consortia might be isolated and propagated in a customized way. Preserving microbial viability and normalizing the process of sampling and delivery is critical in the whole microbiome pipeline^1^. To this end, the fecal microbiota needs to be maintained unaltered and representative of the original microbial populations harbored by the host.

A microbial subpopulation concerned by our approach is the species *F. prausnitzii* one of the major butyrate producers of the large intestine and a EOS microorganism^15,16^. Most importantly, reduction in the relative abundance of this bacterium is associated to the dysbiosis associated to type 2 diabetes, inflammatory bowel disease or other chronic inflammatory conditions^16–20^. Recent research supports the anti-inflammatory mechanism of action of *F. prausnitzii* by the secretion of different molecules^20–22^. Very interestingly, butyrate-producers seems to be host-specific, so keeping its biodiversity by avoiding the toxic effect of oxygen during sampling, will allow to envisage the development of personalized, next-generation probiotics in case of future neediness.

GutAlive is also useful for heterologous FMT, which has been used successfully during the last years to eradicate the multidrug-resistant human pathogen *Clostridium difficile*, restoring at the same time a healthy microbiome. FMT reflects both the outstanding applications of FMT and the relevance of using complete microbiota to achieve a therapeutic effect^23^. The use of devices such as GutAlive will grant to normalize the process on sample collection and delivery to ensure the microbial viability, allowing to store aliquots of healthy microbiota configurations. The storage of healthy viable microbiota will open new avenues to be used in personalized microbiome restauration therapies when an individual suffers a disease or an extensive medical treatment (e.g. long-term antibiotic administration or chemotherapy) that devastate the microbiota. Personalized biotherapeutic applications include aFMT and formulations of personalized probiotics mix designed specifically not only to the individual, but rather to the particular microbiota-associated alteration^24^.

A recent study has shown outstanding results regarding microbiome restorage using aFMT after extensive antibiotic treatment^4^. Microbiota richness measured as an alpha diversity index was similar to the control after eight days in the animal model and just after one day in a group of human volunteers following aFMT. Interestingly, the same work showed that a multispecies probiotic product was less efficient in terms of microbiota recovery as deduced from the alpha diversity index. Although freezing stool samples (either at −20 °C or −80 °C) immediately following collection, is still considered the gold standard of feces collection for microbiota studies, notably if a metagenomic approach is considered, our technology will allow standardizing the collection and transport of the samples provided by thousands of different people, as temperature during those two process has little effect over the viability of the major obligate anaerobe bacteria present in the samples. Care should be taken in the sense that our approach is reflecting a real procedure, in which the user samples the fecal specimen at home being he/she is responsible to ship the sample by courier tback to our lab. For this we have estimated an average time of 5 h and, which is considered as baseline. Since microbiota was not tested for viability at time zero (just after deposition), we can afirm that GutAlive is better than a conventional stool container, but we cannot strictly say that GutAlive maintain all the anaerobic bacteria of the sample, as some microorganisms might be lost during the transport of the simple to the lab.

Microbiome analysis and more importantly, microbiome restoration therapies require the standardization of protocols and materials, including fecal sampling devices that ensure the viability of the fecal microbiota upon collection, transport and storage prior to any analysis or treatment. However, current and past international initiatives have been focused on the standardization of protocols for DNA sequence analysis and processing, whereas protocols for sample collection, transport and storage have received little attention^25,26^.

## Conclusions

GutAlive allowed maintaining the viability of obligate anaerobes and higher levels of diversity when compared to the conventional stool collection device. The capability of maintaining fecal microbiota viability open new avenues normalizing microbiome analysis and further steps like isolation and storage of viable microbiotas, critical for developing therapeutic and personalized strategies to improve human health, based on intestinal microorganism consortia. Personalized and viable microbiota storage will be in the near future a source for developing new therapeutic products based on defined and personalized microbiotas to be applied in microbiome restoration therapies such as aFMT.

## Material and Methods

### Sample collection and patient recruitment

The results showed in this work were obtained from the feces of six healthy donors (three male and three females; age range between 33 and 48 years old). Subject recruitment was carried out at the Dairy Research Institute of Asturias (Villaviciosa, Spain). Four different stool samples from each individual were used for each experiment. The exclusion criteria were: having undergone medical treatment with antibiotics or glucocorticoids during the previous three months. Volunteers were informed of the objectives of the study and all samples were collected and analyzed with fully informed written consent from all participants involved in the study. The samples were anonymized using an alphanumeric code. Stools were collected by the final users, which were responsible to keep the samples either at room temperature or refrigerated and transport them to our facilities. The first time interval, 5 hours, is considered as the average transport time a participant would employ to collect and transport the sample to the lab, and it is thus considered as our zero time. In order to understand the toxic effect of oxygen over bacteria viability, feces were sample either using GutAlive (http://www.microviable.com) or a conventional stool collection device (Deltalab, ref. 409726).

### Strains and culture conditions

*B. bifidum* LMG11041 was grown on selective media MRSc [De Man, Rogosa and Sharpe agar (VWR chemicals, USA)] supplemented with 0.25% (w/v) cysteine (VWR chemicals) and *F. prausnitzii* M21/2 in modified Brain Hearth Infusion (mBHI; Oxoid, UK). BHI medium was prepared following manufacturer instructions plus 5 g/L yeast extract (Oxoid), 0.05% (w/v) cysteine, 0.1% maltose (w/v) (Sigma-Aldrich, Germany), and 0.1% cellobiose (w/v) (Sigma-Aldrich).

For fecal microbiota viability and diversity assays 4 different media were used: GAMc, Gifu Anaerobic Medium (HyServe GmbH, Germany), plus 0.25% (w/v) cysteine; modified BHI supplemented (mBHIb) or not (mBHI) with 10% (v/v) defibrinated horse blood (Fisher scientific, USA) and Anaerobe Basal Medium (Oxoid) supplemented with 10% (v/v) defibrinated horse blood (ABMb). Differential colonies were selected attending to characteristics related mainly to their size, shape, elevation, margin, appearance, consistency, color and opacity.

### Bacteria identification and 16S rRNA gene sequencing

Bacterial diversity was calculated as the differential colonies recovered with GutAlive and the standard device, based on colony phenotype. In addition, we applied the Margalef Species Richness Index for estimating alpha diversity^27^, as it takes into consideration the number of different species compared to the total number of individuals: in this case number of different colony phenotypes with respect to the total colony counts. All the colonies recovered in the different growth media were identified by sequencing their 16S rRNA gene. Total DNA extracted using a general chloroform-based protocol and used as template to amplify the 16S rRNA gene by PCR with 16S universal primers 27 F (5′-AGAGTTTGATCCTGGCTCAG-3′) and 1492 R (5′GGCTACCTTGTTAGCGACTT-3′). Reaction was carried out using Dreamtaq Hot Start master Mix (Fisher Scientific). The amplicons were sent for sequencing to the DNA sequencing facilities of Macrogen (Madrid, Spain). The 16S rDNA sequences were further analyzed by BLASTn searches against the nr NCBI database. Alignment rules for taxonomical assessment were the defaults for BLASTn and the top blast hit on nr NCBI database.

### Genome sequencing and annotation

Genomic DNA from the MT126 and MT139 isolates was purified using DNeasy blood and tissue kit (Qiagen, Germany) and genome sequencing were determined using a 250–290 paired-end libraries with Illumina MiSeq Sequencing System (Illumina, USA) at GenProbio SRL (Parma, Italy). Genome assemblies were achieved with PATRIC 3.5.23 online resource (https://www.patricbrc.org/app/Assembly)^28^. Paired-end read files were uploaded and SPAdes assembly strategy was selected. Automatic annotation of the Open Reading Frames (ORFs) were carried out by RAST (Rapid Annotation using Subsystem Technology) online resource^29^.

### *Faecalibacterium* pangenome

*Faecalibacterium* sp. pangenome was computed using the Roary software, following the pipeline described in the online manual (https://sanger-pathogens.github.io/Roary/)^30^. Briefly, GFF3 files were firstly obtained using the genome sequences as input of Prokka^31^. Pangenome was computed using the “roary -e–mafft -p 8 *.gff” command and analysed using the different options available for the “query_pan_genome” command.

### In silico DNA-DNA hybridization

In silico DNA-DNA hybridization (DDH) experiments were estimated using the Genome-to-Genome Distance Calculator (GGDH), available at http://ggdc.dsmz.de/ ^32^. This was performed for two sequence groups, the first comprising 27 *F. prausnitzii* complete genomes, and the second with 43 complete genomic sequences, corresponding to representatives of species within the order *Clostridiales*, family *Ruminococcaceae*. The complete genome of the isolate MT126 was included in the first group, whereas that of isolate MT139 was incorporated into the second. With the exception of these two genomes, sequenced in this work, the rest of genomes were retrieved from the NCBI using the genome browser (https://www.ncbi.nlm.nih.gov/genome/genomes).

For DDH estimates, we used the formula #2 (recommended by the GGDC) in which the number of identities is divided by the high-scoring segment pair (HSP) length. The result of this formula is a distance which is transformed to values analogous to wet DDH through the application of a generalized linear model trained on an empirical reference dataset.

### Phylogeny reconstruction based on 16S rRNA gene sequences

Forty two nucleotide sequences corresponding to the closest hits obtained with the fecal isolate MT139 16S rDNA sequence were obtained using BLASTn, (excluding uncultured/environmental sample sequences) These sequences were downloaded in multifasta format and kept for further analysis: NR_144748.1, LT558849.1, NR_147378.1, LT598596.1, LT558850.1, LT598578.1, NR_147398.1, LN881593.1, NR_027558.1, NR_044425.1, LT854295.1, LT576408.1, NR_158111.1, KY777734.1, LT598551.1, NR_029355.1, NR_115230.1, NR_074399.1, NR_102884.1, LT934440.1, LN881596.1, NR_074333.1, NR_115307.1, NR_044624.1, NR_042930.1, NR_144727.1, LN866991.1, NR_144736.1, LN998059.1, LT960605.1, NR_114789.1, LN870298.1, N870316.1, NR_156911.1, NR_104846.1, NR_028997.1, NR_028961.1, NR_151900.1, KM098109.2, NR_029315.1, NR_029313.1, LT631513.1. The 16S rDNA sequence corresponding to isolate MT139 was extracted from its assembly genome s.

A multiple alignment of nucleotide sequences was obtained using ClustalW2^33^ and manually corrected where necessary. The final multiple alignments were systematically filtered for gaps and poorly aligned positions, which are known to introduce phylogenetic noise, using Gblocks^34^. Phylogenies based on 16S rRNA gene sequences were inferred using the Neighbor-Joining (NJ) method (bootstrap = 5,000). The evolutionary distances were computed using the Kimura 2-parameter method and were expressed in the units of the number of base substitutions per site. The rate variation among sites was modeled with a gamma distribution (shape parameter = 1). All positions containing gaps and missing data were eliminated. There were a total of 1114 positions in the final dataset. Evolutionary analyses were conducted in MEGA7^35^.

## Declarations

### Ethics approval and consent to participate

Ethics approval for this study (reference code 185/18) was obtained from the Regional Ethics Committee for Clinical Research (*Comité de Ética de la Investigación del Principado de Asturias*) in compliance with the Declaration of Helsinki. Samples used in this study were obtained from anonymous donors. Informed consent was obtained from all volunteers.

## Supplementary information

Supplementary File 1

Supplementary File 2

Supplementary File 3

Supplementary Figure 1

## Data Availability

The datasets generated and analyzed in the current study were deposited at the GenBank Database (16S rDNA sequences, accessions: MK121818-MK121885). The genomes of the isolates MT126 and MT139 are publicly available at the PATRIC website (https://www.patricbrc.org/).
