# Supplementary material for: Filling the gap between collection, transport and storage of the human gut microbiota
Source: Sci Rep. 2019 Jun 6;9:8327. doi: 10.1038/s41598-019-44888-8 (PMC6554407; doi:10.1038/s41598-019-44888-8)
Supplement: Supplementary file 4 — Supplementary Figure 1 [file 41598_2019_44888_MOESM4_ESM.pdf]

**Supplementary figure 1**: Dendrogram created using the presence/absence of accessory genes in binary format. *In silico* DDH distances are shown in blue as percentages, next to the label. Most of the branches are collapsed for a better visualization of the tree.

Filling the gap between collection, transport and storage of the human gut microbiota.

Noelia Martínez, Claudio Hidalgo-Cantabrana, Susana Delgado, Abelardo Margolles, Borja Sánchez3

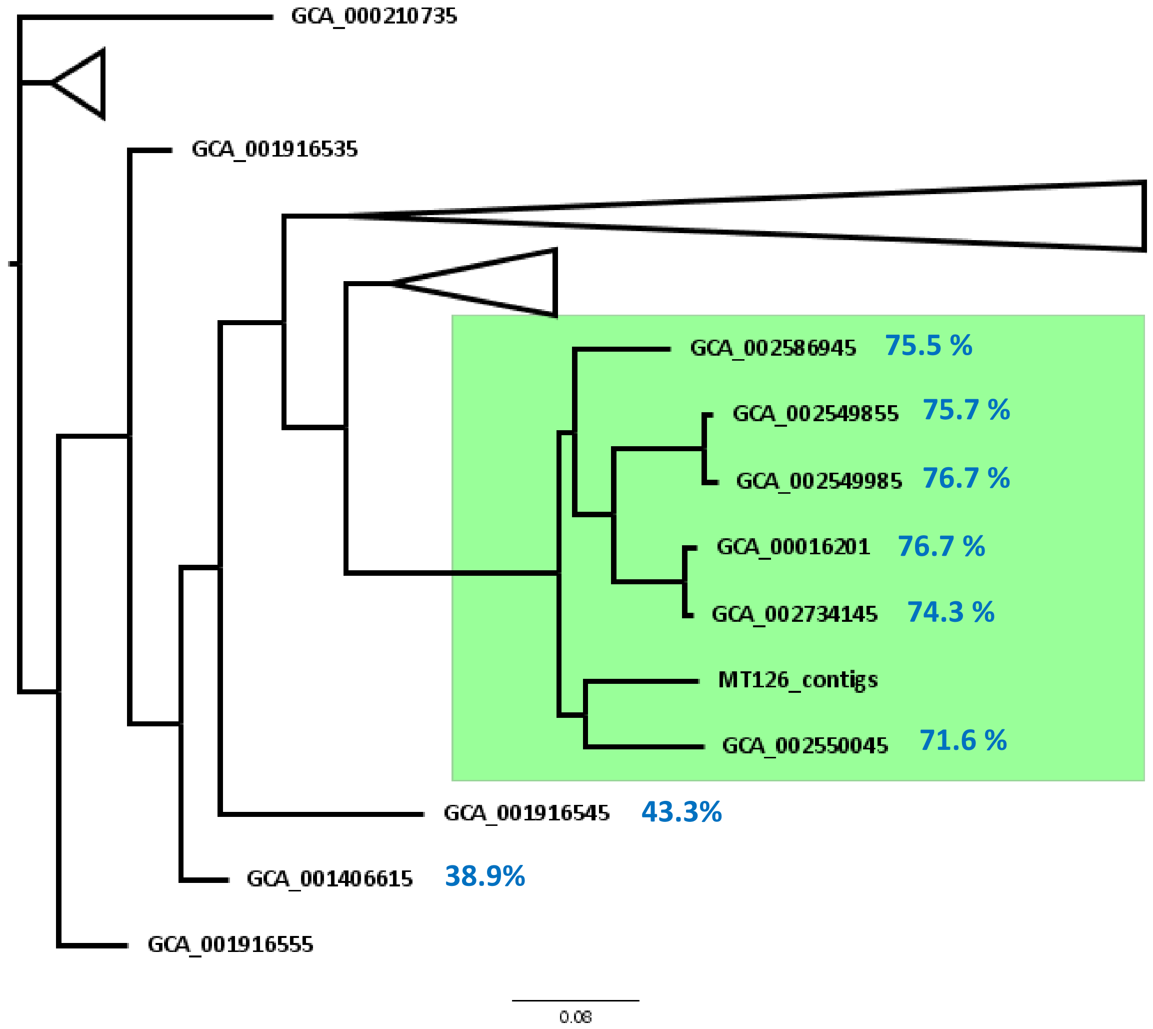
GCA_000210735
GCA_001916535
GCA_002586945 75.5 %
GCA_002549855 75.7 %
GCA_002549985 76.7 %
GCA_00016201 76.7 %
GCA_002734145 74.3 %
MT126_contigs
GCA_002550045 71.6 %
GCA_001916545 43.3%
GCA_001406615 38.9%
GCA_001916555
0.08
